# Efficient parameter estimation for ODE models of cellular processes using semi-quantitative data

**DOI:** 10.1093/bioinformatics/btae210

**Published:** 2024-06-28

**Authors:** Domagoj Dorešić, Stephan Grein, Jan Hasenauer

**Affiliations:** Life and Medical Sciences (LIMES) Institute, University of Bonn, 53113 Bonn, Germany; Institute of Computational Biology, Helmholtz Zentrum München – German Research Center for Environmental Health, 85764 Neuherberg, Germany; Life and Medical Sciences (LIMES) Institute, University of Bonn, 53113 Bonn, Germany; Life and Medical Sciences (LIMES) Institute, University of Bonn, 53113 Bonn, Germany; Institute of Computational Biology, Helmholtz Zentrum München – German Research Center for Environmental Health, 85764 Neuherberg, Germany; Center for Mathematics, Technische Universität München, 85748 Garching, Germany

## Abstract

**Motivation:**

Quantitative dynamical models facilitate the understanding of biological processes and the prediction of their dynamics. The parameters of these models are commonly estimated from experimental data. Yet, experimental data generated from different techniques do not provide direct information about the state of the system but a nonlinear (monotonic) transformation of it. For such semi-quantitative data, when this transformation is unknown, it is not apparent how the model simulations and the experimental data can be compared.

**Results:**

We propose a versatile spline-based approach for the integration of a broad spectrum of semi-quantitative data into parameter estimation. We derive analytical formulas for the gradients of the hierarchical objective function and show that this substantially increases the estimation efficiency. Subsequently, we demonstrate that the method allows for the reliable discovery of unknown measurement transformations. Furthermore, we show that this approach can significantly improve the parameter inference based on semi-quantitative data in comparison to available methods.

**Availability and implementation:**

Modelers can easily apply our method by using our implementation in the open-source Python Parameter EStimation TOolbox (pyPESTO) available at https://github.com/ICB-DCM/pyPESTO.

## 1 Introduction

The use of mechanistic mathematical models has greatly contributed to the understanding of biological processes at the cellular (Kitano 2002, Schöberl *et al.* 2009), patient (Fey *et al.* 2015, Hass *et al.* 2017) and population level (Giordano *et al.* 2020, Zhao and Chen 2020). In particular, mechanistic ordinary differential equation (ODE) models are used for a broad spectrum of applications, ranging from cellular signaling, metabolism, and gene regulation over pharmacokinetics and -dynamics to the spread of diseases. However, ODE models often contain parameters that cannot be measured directly. Instead, the parameters have to be estimated from experimental data (Mitra and Hlavacek 2019). This is commonly achieved by numerical optimization of an objective function, which quantifies how well the model simulations fit the given experimental data, such as the likelihood function.

**Figure 1.**
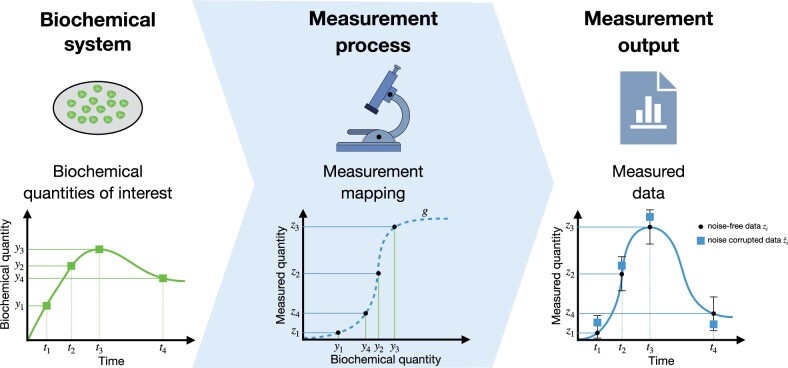
A nonlinear measurement mapping. (Biochemical system) True values of a biochemical quantity of interest ${\left\{(t_{i},y_{i})\right\}}_{i=1}^{4}$. (Measurement process) A measurement process can introduce unknown nonlinear data mappings (dashed blue line). In that case, a mapping function transforms the biochemical quantities ${\left\{y_{i}\right\}}_{i=1}^{4}$ and yields nonlinearly mapped measured quantities ${\left\{z_{i}=g(y_{i})\right\}}_{i=1}^{4}$. (Measurement output) The measurement quantities ${\left\{z_{i}\right\}}_{i=1}^{4}$ are corrupted by noise, resulting in a noise-corrupted dataset $D={\left\{(t_{i},{\tilde{z}}_{i})\right\}}_{i=1}^{4}$

The experimental data used for parameter estimation are collected using a broad spectrum of experimental techniques. For example, early studies in the field of systems biology employed well-calibrated Western blot experiments and performed an in-depth assessment of the mapping of concentration to measured intensities (Kreutz *et al.* 2007). In this case, the data were ensured to fall within the linear regime of the experimental technique, and often even absolute quantification was performed. However, many, even state–of–the–art, measurement techniques do not ensure a linear relationship between the abundance of the biochemical quantities of interest and the measured output (Fig. 1). Well-known examples include fluorescence microscopy data such as Förster resonance energy transfer (FRET) data (Birtwistle *et al.* 2011), optical density (OD) measurement (Stevenson *et al.* 2016) and imaging data for certain stainings (Pargett *et al.* 2014). In addition, many experimental techniques suffer from lower limits of detection and/or saturation effects.

Quantitative data are easy to use for the parameterization of ODE models and the same holds for data that are collected in the linear regime of measurement devices. This is showcased in a large number of published articles [see, e.g. (Hass *et al.* 2019) for a collection of models and datasets]. In fact, there are custom methods for experimental data for which a linear mapping with unknown scaling and offset parameters can be assumed (Loos *et al.* 2018, Schmiester *et al.* 2019). If the linearity assumption is not fulfilled, it is usually assumed that the mapping from biochemical quantities of interest to measured output is monotone. This monotonicity ensures that the ordering is preserved and allows the use of approaches for ordinal data, such as the optimal scaling method (Shepard 1962). For ODE models, this approach has recently been accelerated by using a reformulation of the optimization problem (Schmiester *et al.* 2020) and gradient information (Schmiester *et al.* 2021b). However, in this approach, all quantitative information is discarded and the defined objective function is not based on probabilistic grounds, disallowing any uncertainty analysis.

In this manuscript, we introduce a spline-based approach to use semi-quantitative data—which are obtained using an experimental technique with a nonlinear but monotone mapping—for parameter estimation. We assume that the measurement mappings are increasing monotonically. This is frequently observed in experiments: values with a larger measurement value are assumed to correspond to larger biochemical quantities of interest. The method reconstructs the unknown mapping function using a statistically coherent formulation that facilitates uncertainty analysis. We demonstrate the credibility of the proposed approach as a tool for uncovering measurement mapping shapes. To illustrate the parameter inference capabilities of the method, we benchmark its performance with a collection of published models. Furthermore, we derive formulas for the analytical calculation of the gradients of the objective function in hierarchical optimization. To evaluate this optimization framework, we compare its efficiency with alternative approaches.

## 2 Materials and methods

### 2.1 Mechanistic modeling of biological systems

We consider models of biological processes based on systems of ODEs:

(1) $$\dot{x}(t,\theta )=f(x(t,\theta ),\theta ,t),\;x(t_{0},\theta )=x_{0}(\theta )$$

in which the temporal evolution of the state variables $x(t,\theta )\in R^{n_{x}}$ is determined by the vector field $f:R^{n_{x}}\;\times \;R^{n_{\theta }}\;\times \;R_{+}\to R^{n_{x}}$ with the unknown mechanistic model parameters $\theta \in R^{n_{x}}$. State variables can, e.g. describe protein concentrations at the level of cellular processes or groups of individuals at the level of population modeling. The parameters θ usually consist of kinetic rate constants and initial species conditions. In cellular models, often not all metabolites are measurable, or in some cases only the sum of concentrations of multiple metabolites can be observed. These measured properties of a model are its observables, denoted as $y\in R^{n_{y}}$,

(2) $$y=h(x,\theta ),\;\tilde{y}=y\;+\;\varepsilon \;\text{with}\;\varepsilon ∼N(0,\sigma ),$$

in which $h:R^{n_{x}}\;\times \;R^{n_{\theta }}\to R^{n_{y}}$ denotes the observation map which models the dependence of the observables on the model state variables and unknown mechanistic parameters, $\sigma \in R_{+}^{n_{t}}$ is a noise parameter, and $\tilde{y}\in R^{n_{y}}$ are noise-corrupted measurements. The dimensionalities of the state, parameter, and observable vector are denoted by $n_{x}$, $n_{\theta }$, and $n_{y}$, respectively. The number of time points is denoted by $n_{t}$.

#### 2.1.1 Linear semi-quantitative (relative) observables

Most measurement techniques provide only relative information on the biochemical quantity of interest. In this case, to obtain values comparable to the measured quantities, those observables need to be rescaled by scaling factors *a* and offsets *b*. This is the case, for instance, for well-calibrated Western blot measurements, where the modeled protein concentrations have to be rescaled to be comparable to the optical density measurements. Most often, an additive Gaussian distributed noise model is assumed. Then the full relationship between measured and biochemical quantities of a relative observable is given by:

(3) $${\tilde{z}}_{ik}=\underset{:=g_{i}(h_{i}(x(t_{k},\theta ))}{\underset{︸}{a_{i}h_{i}(x(t_{k},\theta ),\theta )\;+\;b_{i}}}\;+\;\varepsilon_{ik},$$

$$\begin{array}{c} \text{with}\;\varepsilon_{ik}∼N(0,\sigma_{ik}^{2}),\;g_{i}(x)=a_{i}\cdot x\;+\;b_{i} \end{array}$$

in which *i* is the observable index, *k* is the time index, and $g_{i}:R\to R$ is a scaled and offset (affine) measurement mapping from the *i*th observable, $h_{i}(x(t_{k},\theta ))$, to its measurement. The scaling factors, offsets, and noise parameters of the *i*th observable are denoted as $a_{i}\in R$, $b_{i}\in R$, and $\sigma_{i}\in R_{+}^{n_{t}}$, respectively. These parameters are often unknown and need to be estimated along with all other unknown parameters of the model.

#### 2.1.2 Nonlinear semi-quantitative observables

In some cases, the measurement process induces a nonlinear mapping between the biochemical quantities of interest and the measured quantities. A common example is FRET measurements, which will be further investigated in the Section 3. Assuming an additive Gaussian distributed noise model the relationship is given by:

(4) $${\tilde{z}}_{ik}=g_{i}(h_{i}(x(t_{k},\theta ),\theta ))\;+\;\varepsilon_{ik},$$

$$\begin{array}{c} \text{with}\;\varepsilon_{ik}∼N(0,\sigma_{ik}^{2}) \end{array}$$

in which $g_{i}:R\to R$ is a nonlinear measurement mapping from the *i*th observable, $h_{i}(x(t_{k},\theta ))$, to its measurement. The form and parameterization of nonlinear measurement mappings $g_{i}(h_{i}(x,\theta ))$ are application-dependent. We provide examples in the Section 3. Measurement mappings are often unknown and need to be modeled in some way.

**Figure 2.**
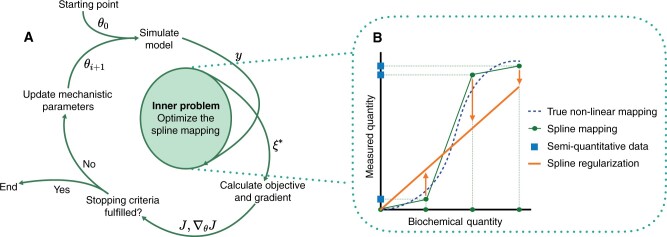
Illustration of the spline estimation approach. (A) Model mechanistic parameters θ are iteratively updated during parameter estimation. For each vector of trial parameters, the model is simulated to obtain the simulation *y*. Then, the spline parameters $\xi^{*}$ are optimized and used to calculate the objective function *J* and its gradient $\nabla_{\theta }J$. These are then passed on to obtain the next trial parameter vector, or the optimization is halted. (B) The spline (green) enables mapping of the simulation of the model *y* (biochemical quantities) to the measurement axis. This allows for the definition of a likelihood objective function. In the inner problem, this objective function is minimized with respect to the spline parameters to obtain optimal spline parameters $\xi^{*}$. The spline is additionally regularized by the distance to the linear mapping (orange)

As this study considers the data-driven uncovering of measurement mappings, we select a class of approximations. Specifically, we consider the approximation of measurement mappings $g_{i}(h_{i}(x,\theta ))$ with monotone piecewise linear splines $s_{i}:R\;\times \;R^{n_{\xi }^{i}}\to R$, in which *i* is the observable index. For the simplicity of further calculation, we parameterize the splines using the differences between the heights of neighboring spline knots ${\left\{\xi_{ij}\right\}}_{j=1}^{n_{\xi }^{i}}$:

(5) $$\begin{array}{c} s_{i}(x,\xi_{i}):=\{\begin{array}{cc} \frac{x}{c_{i1}}\cdot \xi_{1}, & x\;\leq \;c_{i1}\\ \frac{x\;-\;c_{i(j\;-\;1)}}{\Delta_{c}^{i}}\xi_{ij}\;+\;\sum_{l=1}^{j\;-\;1}\xi_{il}, & c_{i(j\;-\;1)}\;\leq \;x\;\leq \;c_{ij}\\ \xi_{in_{\xi }^{i}}, & x\;>\;c_{in_{\xi }^{i}} \end{array} \end{array}$$

in which ${\left\{c_{ij}\right\}}_{j=1}^{n_{\xi }^{i}}$ are the knot bases, and $n_{\xi }^{i}$ is the number of spline knots for the *i*th observable. Since $g_{i}$ is monotone, we constrain the spline parameters to be positive $\xi_{i,j}\;\geq \;0$ for all $j=1,\ldots ,n_{\xi }^{i}$. We regularized the spline by adding a penalty term to the objective function to promote linearity (Fig. 2B), which greatly improved the convergence of the estimation. For details on the definition of the spline, the distribution of the knot bases, and spline regularization, we refer to the first section of the supplementary materials.

The spline allows us to link the measured and biochemical quantities of the nonlinear monotone observable:

(6) $${\tilde{z}}_{ik}=s_{i}(h_{i}(x(t_{k},\theta ),\theta ),\xi_{i})\;+\;\varepsilon_{ik},$$

$$\begin{array}{c} \text{with}\;\varepsilon_{ik}∼N(0,\sigma_{ik}^{2}). \end{array}$$

The model dataset $D={\left\{{\left\{(t_{k},{\tilde{z}}_{i_{k}})\right\}}_{k=1}^{n_{t}}\right\}}_{i=1}^{n_{y}}$ consists of observations of all model observables at time-points ${\left\{t_{k}\right\}}_{k=1}^{n_{t}}$. We denote the dataset of the *i*th observable as $D_{i}={\left\{(t_{k},{\tilde{z}}_{i_{k}})\right\}}_{k=1}^{n_{t}}$.

### 2.2 Parameter estimation

For a dataset D consisting of independent observations of quantitative, linear semi-quantitative, and/or nonlinear semi-quantitative observables, the negative log-likelihood objective function is commonly defined as:

(7) $$J(\theta ,\psi )=\sum_{i=1}^{n_{y}}J_{i}(\theta ,\psi_{i})=\sum_{i=1}^{n_{y}}\;-\;\text{log}\;L_{D_{i}}(\theta ,\psi_{i})$$

in which $\psi_{i}$ are the observable parameters of the *i*th observable: for a relative observable these are scaling $a_{i}$ and offset $b_{i}$, while for a nonlinear semi-quantitative observable they are the spline parameters $\xi_{i}$. Minimizing the objective function, we obtain the maximum likelihood estimate (MLE): $\theta^{*},\psi^{*}=\underset{\theta ,\psi }{\arg\min}J(\theta ,\psi )$.

#### 2.2.1 Hierarchical optimization problem and analytical gradients

The objective function minimization can be executed jointly in all mechanistic parameters θ and observable parameters ψ. However, this leads to a high-dimensional optimization problem and long computation times. Alternatively, the optimization problem can be separated hierarchically (Fig. 2A):

(8) $$\underset{\theta }{\min}\;J(\theta ,\psi^{*}(\theta ))$$

(9) $$\begin{array}{c} s.t.\;\{\begin{array}{c} \psi_{i}^{*}(\theta )=\underset{\psi_{i}}{\arg\min}J_{i}(\theta ,\psi_{i}) \end{array}\text{for}\;i=1,\ldots ,n_{y}. \end{array}$$

In the outer optimization problem (8), we estimate the mechanistic parameters θ, and in the inner optimization problems (9) we estimate the observable parameters $\psi_{i}$ of each observable. For nonlinear semi-quantitative observables, the inner problem is additionally constrained by the positivity of spline parameters. Since the objective function can be additively separated into components ${\left\{J_{i}\right\}}_{i=1}^{n_{y}}$ depending on the observable parameters of a single observable $\psi_{i}$, the inner optimization problem is a set of $n_{y}$ small inner optimization problems (9). For relative observables, the inner problems (9) can be solved analytically (Schmiester *et al.* 2019). For nonlinear semi-quantitative observables, they still need to be numerically minimized, but the inner problems are convex and thus easy to minimize. Second, the gradient of the objective function can be calculated analytically. We formulate and prove the two statements above in Theorems 1 and 2 of the supplementary materials.

### 2.3 Confidence region of a parameter vector θ

Here, we define what it means for a parameter vector to lie in a confidence region of a certain significance. We do this using the likelihood-ratio test in which we define the corresponding test statistic as

(10) $$\Lambda (\theta )=\;-\;2\;\text{log}\;\left(\frac{L_{D}(\theta )}{\underset{\theta }{\sup}\{L_{D}(\theta )\}}\right)=2(J(\theta )\;-\;J(\theta^{*})).$$

In the asymptotic case of a large number of data points, the test statistic converges to a chi-square distribution $\chi_{\text{df}}^{2}$ with $\text{df}=n_{\theta }$ degrees of freedom [see Tönsing *et al.* (2023) for more details]. Then we define the confidence region of significance α as

(11) $${\text{CR}}^{\alpha }=\{\theta |\Lambda (\theta )\;\leq \;\Delta_{\alpha }\}$$

in which $\Delta_{\alpha }$ is the αth percentile of the $\chi_{n_{\theta }}^{2}$ distribution.

### 2.4 Scalability and complexity of the proposed method

The inner optimization objective functions $J_{i}(\theta ,\psi_{i})$ for semi-quantitative observables are convex and self-concordant. Thus, their numerical optimization using barrier methods scales mostly with the number of inequality constraints in the inner problem (Boyd and Vandenberghe 2004), i.e. with the number of spline parameters $\xi_{i}$ of the spline mapping (5). In most applications, this number should be set to a small value (5–10), as this already provides sufficient modeling capacity for most nonlinear measurement mappings and also reduces overfitting. Therefore, in larger ODE systems, the optimization of inner problems for semi-quantitative observables constitutes a small part of computational complexity and the proposed method scales linearly in the number of semi-quantitative observables.

### 2.5 Benchmark models

For the evaluation of the proposed method, we consider one exemplary model and four published models that were previously developed and calibrated for different biological systems (Table 1). As the published models originally did not contain nonlinear semi-quantitative observables, we generated synthetic data at the same time points, chose nonlinear monotone measurement mappings, applied them to the observables, and corrupted them with the same type of noise as in the original model. For details on the synthetic data generation, chosen nonlinear measurement mappings, and model structure, we refer to the second section of the supplementary material.

**Table 1.** Benchmark models.^a^

Model	$n_{x}$	$n_{\theta }$	$n_{y}$	|D|	Reference
T1	2	4	1	12	Birtwistle *et al*. (2011)
M1	8	6	3	48	Boehm *et al*. (2014)
M2	7	9	1	23	Rahman et al. (2016)
M3	8	18	1	58	Elowitz and Leibler (2000)
M4	14	18	8	205	Raia *et al*. (2011)

a By |D| we denote the cardinality of the dataset.

## 3 Results

### 3.1 The proposed method uncovers measurement mapping for FRET probe activation

**Figure 3.**
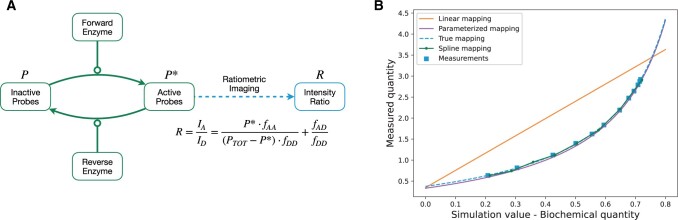
A model of FRET probe activation. (A) A forward enzyme catalyzes the activation of inactive FRET probes *P*, and a reverse enzyme catalyzes the conversion of an active probe $P^{*}$ into the inactive state. Active probe concentration can be observed via ratiometric imaging. The measurement mapping of this process is highly nonlinear. $f_{AA}$ and $f_{AD}$ are fractions of the acceptor and donor emissions that the acceptor channel captures, respectively. $f_{DD}$ is the fraction of donor emissions that the donor channel captures. (B) Comparison of the estimation of the measurement mapping using a linear function, proposed spline mapping, and parameterization of the true mapping. For all three models, we performed 1000 local optimizations. Depicted are the estimated mappings closest to the true mapping of starts with mechanistic parameters in the 95% confidence region. We show the synthetic noise-corrupted data used in all model optimizations in blue squares

To illustrate an application of the proposed method, we have applied it to a FRET probe activation model introduced by Birtwistle *et al.* (2011). In general, the transition of inactive FRET probes *P* to an active state $P^{*}$ can be represented by the scheme in Fig. 3A. The quantity of interest in this model is the ratio of activated probes to total probes $P^{*}/P_{\mathrm{TOT}}$. The most common way to measure this value is through a measurement technique called ratiometric imaging. Cells are exposed to excitation light from the donor channel, and then fluorescence emission is divided into donor and acceptor channels. The output of ratiometric imaging, R, is the intensity in the acceptor channel, $I_{A}$, divided by the intensity in the donor channel, $I_{D}$. Previous studies have shown that this measured R value can have a highly nonlinear relationship to the fraction of active FRET probes (Birtwistle *et al.* 2011) (Fig. 3A).

One approach of modeling this nonlinear mapping is to parameterize a function of a similar shape and to estimate its parameters. For FRET probe activation it has been shown that the relation between state variables and measurement is of the form:

(12) $$g(P^{*})=\alpha \cdot \frac{P^{*}}{P_{\mathrm{TOT}}\;-\;P^{*}}\;+\;\beta$$

with experiment- and probe-specific parameters α and β. However, this requires prior knowledge of the shape of the measurement mapping. Without such prior knowledge, the measurement mapping has to be inferred. A simple and easy-to-implement approach is to assume that the mapping is linear. This linear approximation can be sufficiently correct if the measurement region is locally linear. For highly nonlinear measurement mappings, this is not true, so one has to resort to more flexible approaches such as spline estimation.

To evaluate how well the three modeling approaches can recover the true measurement mapping, we performed 1000 local optimizations for each and chose the best measurement mapping estimates in the 95% confidence region (Fig. 3B). We found that the reconstruction using spline estimation agrees well with the true measurement mapping. Indeed, it yields similar results to using parametric representations with unknown parameters. In contrast, a linear model for the measurement mapping proved to be insufficiently flexible and resulted in biased reconstruction of the measurement mapping.

Overall, our assessment revealed that, unlike a simple linear approximation for the measurement mapping, a spline-based approximation enables the reconstruction of nonlinear mappings as observed for FRET probe activation.

### 3.2 The spline estimation approach as a tool for uncovering measurement mapping shapes

**Figure 4.**
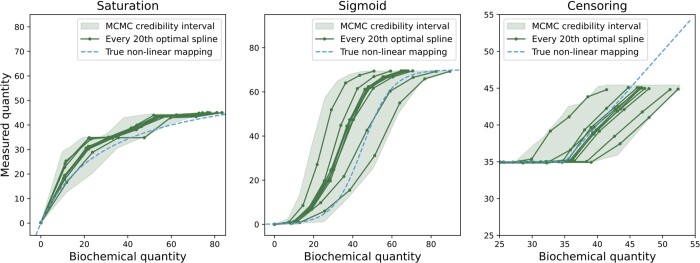
Credibility intervals of the estimated spline mappings. MCMC sampling of the model M1. The model contains different synthetic nonlinear measurement mappings for each of its observables (dashed blue). Splines were estimated for each 500th sample of the MCMC run with which we constructed the credibility intervals of the estimated spline mappings (light green). For visibility, we show only each 20th estimated spline (dark green)

In the previous subsection, we have shown that the estimation of an unknown measurement mapping using a spline can yield results similar to the estimation of a parametric representation. Yet, we only considered a point estimate and did not assess the reliability of the reconstruction. To determine whether the proposed approach provided statistically coherent estimates, we considered model M1 with measurement mappings of various shapes across observables (Fig. 4A–C). Using the resulting dataset, we performed a multi-start optimization ($10^{3}$ runs) to obtain optimal parameters and Markov chain Monte Carlo sampling using an adaptive Metropolis-Hastings algorithm ($10^{5}$ iterations). The resulting chain was thinned by a factor of 500. We computed the optimal spline for each of the remaining samples and, with them, constructed the credibility intervals of the optimal spline mappings.

The inspection of the results confirmed that the optimal splines are qualitatively similar to the measurement mappings used for data generation. Furthermore, more importantly, the measurement mappings used for data generation lie within the credibility intervals. This showcases the reliability of the method as a tool for discovering curve shapes of unknown measurement mappings.

### 3.3 Hierarchical optimization and analytical gradients increase the estimation efficiency

As the method provides reliable estimates for the mappings, we turn to the assessment of the computational cost, which is of high practical relevance. Here, we also wanted to evaluate the impact of (i) reformulation as a hierarchical problem and (ii) the availability of analytical gradients. For this assessment, we considered the published models M1 to M4 with synthetic data with a range of different measurement mappings, as detailed in the second section of the supplementary material. For all models, we performed 1000 local optimizations with equal start points across approaches. We then determined the overall computational cost, the number of function evaluations, and the number of converged starts per computation time.

**Figure 5.**
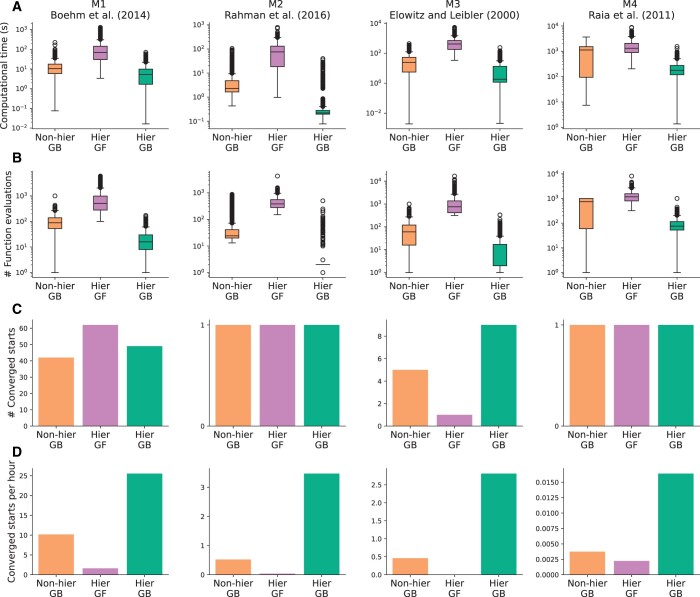
Evaluation of the gradient-based nonhierarchical, gradient-free hierarchical, and gradient-based hierarchical estimation approaches. Models M1–M4 are shown from left to right. (A) Comparisons of computation time. (B) Comparisons of the number of function evaluations. (C) Comparisons of the number of converged starts. Converged starts are defined as the starts with estimated mechanistic parameters within the 95% confidence region. (D) Comparison of the number of converged starts per CPU hour

We found that in general, the proposed hierarchical approach with analytical gradients achieves the best performance (Fig. 5). This appears to be mostly related to a reduction in the computation time, respectively, the number of function evaluations (Fig. 5A and B), while the number of converged starts remains rather similar (Fig. 5C). The number of converged starts per computation time is, for the hierarchical approach with analytical gradients, at least twice as high for the other approaches (Fig. 5D). Interestingly, a hierarchical approach without gradient information does not perform well, and is worse than the nonhierarchical approach with gradient information for all models.

For the proposed hierarchical approach with analytical gradients, the number of converged starts per CPU hour was on average ≈7.96. As the spread between models was large, this finding clearly suggests that the approach is computationally tractable.

### 3.4 Spline approach improves the parameter inference of models with unknown measurement mappings

Our proposed method provides reliable estimates of measurement mappings. Here, we examine whether this leads to good estimates of the mechanistic model parameters. Apart from spline estimation, a generally applicable approach to the integration of semi-quantitative data into parameter estimation is linear estimation of measurement mappings. Thus, we compare the parameter inference of these two approaches in a realistic setting. In addition, for reference, we include the approach of discarding data with unknown measurement mappings. We performed 1000 local optimizations for the application examples M1–M4. We evaluated the impact of an increasing number of unknown measurement mappings by turning quantitative observables into semi-quantitative observables. As a parameter inference metric, we use the mean L2 distance of the estimated to the true mechanistic parameters normalized by the number of mechanistic parameters. For details of the study setting, we refer to the fourth section of the supplementary materials.

**Figure 6.**
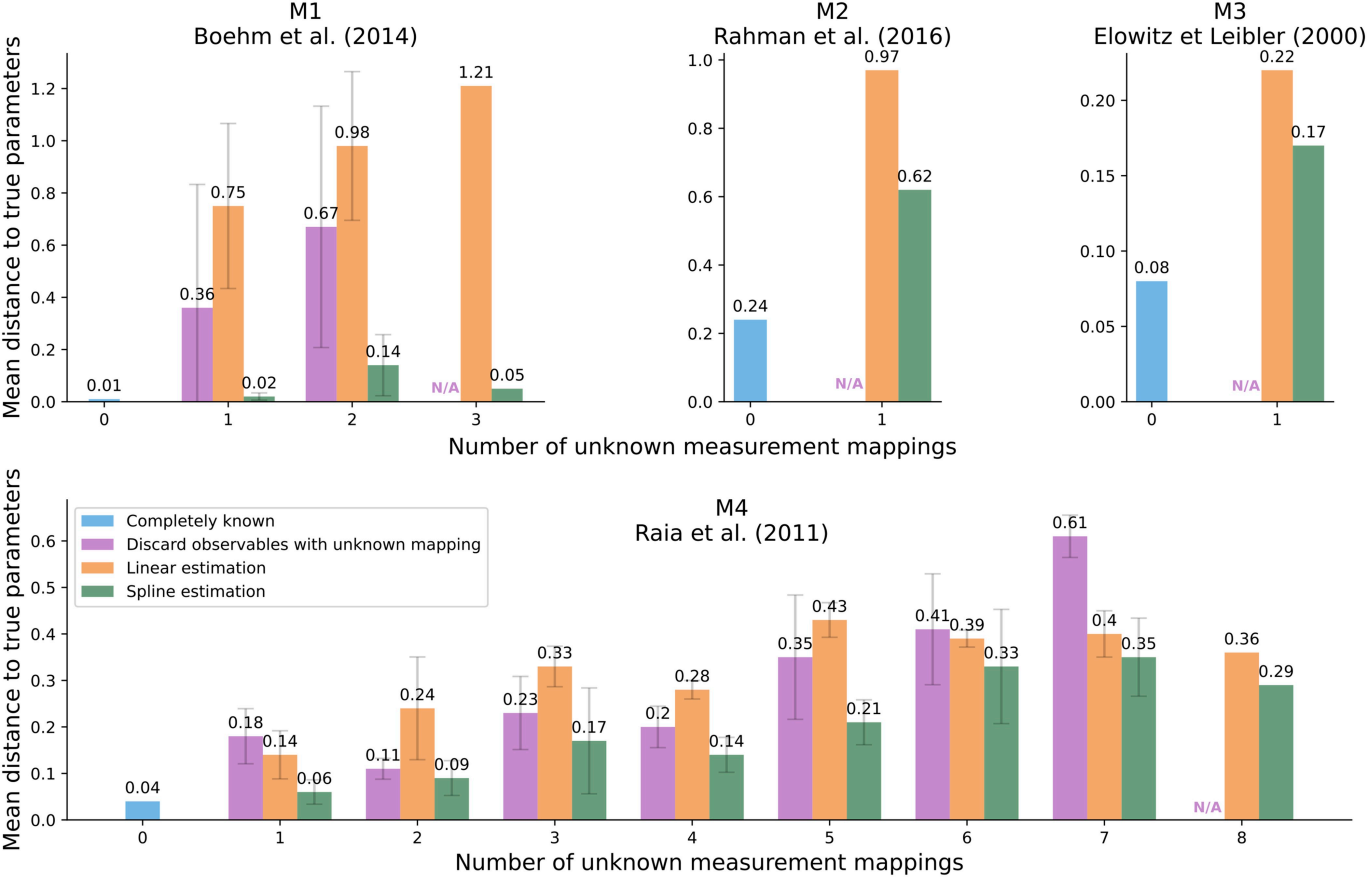
Evaluation of parameter inference across the number of unknown measurement mappings for linear and spline estimation. The parameter inference is measured by the L2 distance of the estimated to the true mechanistic model parameters. On the *x*-axis of each plot, we mark the model variant with a certain number of unknown measurement mappings, ranging from 0 to the number of model observables. The distance for each model variant is normalized by the number of mechanistic parameters and averaged across combinations of known-unknown observables. The best-case scenario for each model M1–M4 is the case of completely known measurement mappings (blue). We compare the distance to the true parameters for the linear (orange) and spline (green) approach for each number of unknown measurement mappings. The approach of discarding the data of observables with unknown measurement mappings (purple) is depicted for reference. This approach is not feasible for the model variants with a maximum number of unknown measurement mappings, as that would involve the removal of all data, so we denote this with N/A

The spline estimation outperforms other approaches (Fig. 6). In general, linear estimation has a stronger bias than variance. We observe this primarily for model M4, as the linear estimation has the smallest standard deviation between approaches (Fig. 6D). In some cases, this even causes the linear estimation to perform worse than the approach of discarding data with unknown mappings. In contrast, the higher flexibility of the spline estimation allows for the general attainment of better parameter estimates. This is the case even for model M4 with eight unknown measurement mappings, for which the spline estimation adds seven times more parameters than the linear estimation. For a small number of unknown measurement mappings, the spline estimation can perform almost equally well as the model with completely known measurement mappings. This showcases that the proposed method yields good estimates of the mechanistic model parameters, especially when the number of unknown measurement mappings is low.

## 4 Discussion

**Figure 7.**
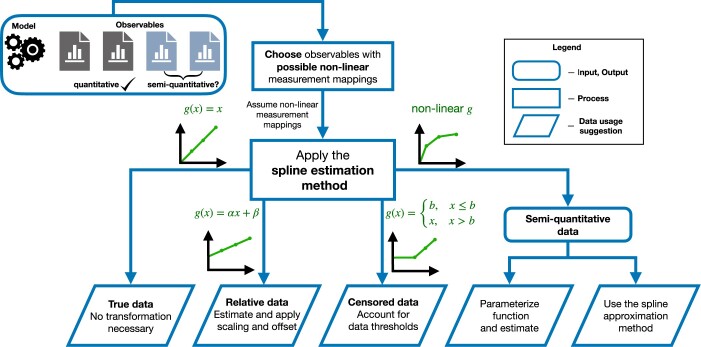
Diagram of application of the spline method to data with possible nonlinear measurement mappings. Estimated optimal splines are depicted in green. Each arrow from the central square is a possible outcome of the nonlinear mapping estimation

Semi-quantitative measurements represent a large portion of the available data that can be used to estimate unknown mechanistic parameters of ODE models. Among others, examples include spatial protein expression assays important in developmental biology, such as chemical staining, fluorescent expression, and immunohistochemistry (Brooks *et al.* 2012). When these are well-controlled, they are expected to linearly transform the true concentration into an image intensity. However, this is not always true: in the case of nonadequate procedural care, hard-to-control outer factors, or insufficient knowledge of the entire experimental system, the transformation function may not be available and can take on a nonlinear form. Here, we address this challenge by introducing a spline-based method for the estimation of unknown nonlinear mappings. The approach can be applied to models with quantitative data for which it is unclear whether the data are truly linear (Fig. 7). Depending on the estimated optimal splines, the data can be deemed to be quantitative, relative, censored, or semi-quantitative, so that an appropriate method can be used. If the estimated optimal spline is nonlinear, one can choose to estimate a parameterized function of a similar shape, or continue using the optimal splines as the measurement mapping. In this way, the method allows for the integration of data previously usable only as qualitative. Furthermore, it can give a clearer understanding of the underlying experimental procedures.

However, one has to be conservative with the number of observables chosen for spline estimation, as it can lead to a large expansion of the parameter space dimension. We evaluated the reliability of this process using an example, showing consistent qualitative measurement mapping shapes. An obvious extension of this approach is the inclusion of symbolic function identification from the estimated optimal splines. This would constitute an automatic parameterization of the unknown measurement mappings.

To increase the method’s efficiency, we employed a hierarchical gradient-based optimization approach. We evaluated its performance and compared it with alternative approaches for four published models with differences in their complexity. This revealed a higher computational efficiency across all models, allowing for faster estimation of parameters for a given model. Further optimization acceleration could be achieved by including adjoint sensitivity analysis (ASA) (Kokotovic and Heller 1967, Fröhlich *et al.* 2017). Although our inner problem is not solved exactly, in Theorem 2 of the supplementary, we show that its gradient contribution is still zero. Thus, existing ASA software implementations from Schmiester et al. (2019) can be used, since the gradient computation is the same as in hierarchical optimization with an exactly solved inner problem. Complementary to this, the derivation of second-order derivatives could further improve the method’s convergence and, with it, its computational efficiency.

The proposed method employs piecewise linear splines to estimate general nonlinear mappings. This was the simplest first-pass option, but, as they are not smooth, they had unavoidable approximation errors. Therefore, it is valuable to explore alternative smooth and flexible parameterized functions. Furthermore, they should retain the convexity of the inner optimization problems and the possibility of analytical gradient calculation. Interesting candidates are the scaled cumulative distribution functions (CDFs) of the beta distribution. They are monotone by definition, parameterized by only three parameters, and with promising flexibility to be able to model most types of measurement nonlinear mappings.

The models for which the method was developed are based on ODE systems primarily because of their widespread prevalence. However, the method can be used more generally. It requires only the model simulations, sensitivities, and the definition of the objective function as a negative log-likelihood. Thus, any model that satisfies these constraints can be incorporated to integrate semi-quantitative data into its estimation of parameters.

In conclusion, we developed and implemented an easy-to-use, computationally efficient framework to uncover unknown nonlinear measurement mappings and to integrate semi-quantitative data into the parameter estimation of ODE models. The approach has a user-friendly implementation in the open-source Python Parameter Estimation TOolbox (pyPESTO). As it is agnostic to the structure of the underlying dynamical model, the method can be applied to models from different research fields, such as physics and engineering.

## Supplementary Material

btae210_Supplementary_Data

## Data Availability

The proposed method is implemented in the open-source Python Parameter Estimation TOolbox (pyPESTO) (schalte2023pypesto). Models M1-M4 were taken from the PEtab benchmark collection (Schmiester et al. 2021a) based on Hass *et al.* (2019). For ODE integration, we used the AMICI Python toolbox (Fröhlich *et al.* 2021). Gradient-based optimization was performed using the fides optimizer (Fröhlich and Sorger 2022) and gradient-free optimization was performed with the SciPy Powell algorithm (Jones *et al.* 2001). Both optimizers were used through the pyPESTO interface with the default optimizer settings. All the code and models used in this study are available from the Zenodo database at https://doi.org/10.5281/zenodo.10568951.
