# Supplementary material for: Efficient parameter estimation for ODE models of cellular processes using semi-quantitative data
Source: Bioinformatics. 2024 Jun 28;40(Suppl 1):i558–66. doi: 10.1093/bioinformatics/btae210 (PMC11211815; doi:10.1093/bioinformatics/btae210)
Supplement: btae210_Supplementary_Data [file btae210_supplementary_data.pdf]

Supplementary information - Efficient parameter estimation for ODE models of cellular processes using semi-quantitative data

Domagoj Drešić,[1,3] Stephan Grein[1] and Jan Hasenauer[1,2,3]

[1]Life and Medical Sciences (LIMES) Institute, University of Bonn, Bonn, Germany
[2]Technische Universität München, Center for Mathematics, Garching, Germany
[3]Helmholtz Zentrum München, German Research Center for Environmental Health, Computational Health Center, Neuherberg, Germany

1 Derivation of analytical gradient formulas

Here, we derive the formulas for the calculation of the analytical gradient of the objective function with respect to the mechanistic parameters in hierarchical optimization.

1.1 Model and spline definition

We consider models based on a system of ODEs

$$\dot{x}(t,\theta) = f(x(t,\theta),\theta), \quad x(t_0,\theta) = x_0(\theta) \tag{1}$$

in which $f : \mathbb{R}^{n_x} \times \mathbb{R}^{n_\theta} \to \mathbb{R}^{n_x}$ is a vector field describing the temporal evolution of the state variables $x \in \mathbb{R}^{n_x}$, and $\theta \in \mathbb{R}^{n_\theta}$ are the unknown mechanistic parameters. The measured properties of the model are its observables $y := h(x,\theta) \in \mathbb{R}^{n_y}$. The dimensionalities of the state, parameter, and observable vector are denoted by n_x, n_θ and n_y, respectively. To simplify the following computations, we assume that our model has only one observable, that is, $n_y = 1$. Later in Subsection 1.5, we show that this can be done without loss of generality. Additionally, let this observable be a non-linear semi-quantitative observable. In other words, there exists a true and unknown non-linear monotone mapping $g : \mathbb{R} \to \mathbb{R}$ such that, assuming an additive normally distributed noise model, the measured data $\{\tilde{z}_k\}_{k=1}^{n_t}$ are given by:

$$\begin{aligned}\tilde{z}_k &= g(h(x(t_k,\theta),\theta)) + \varepsilon_k, \\ &\text{with } \varepsilon_k \sim \mathcal{N}(0,\sigma_k^2)\end{aligned} \tag{2}$$

for time points $\{t_k\}_{k=1}^{n_t}$, in which n_t is the number of observable time-points and $\sigma = (\sigma_k)_{k=1}^{n_t}$ are the noise parameters. Noise parameters are also unknown in some cases and have to be estimated. In the case of such an observable, we refer to $g(h(x(t_k,\theta),\theta))$ as the observable values, and to $y_k(\theta) = h(x(t_k,\theta)$ as the (biochemical, epidemiological, ...) quantities of interest. We

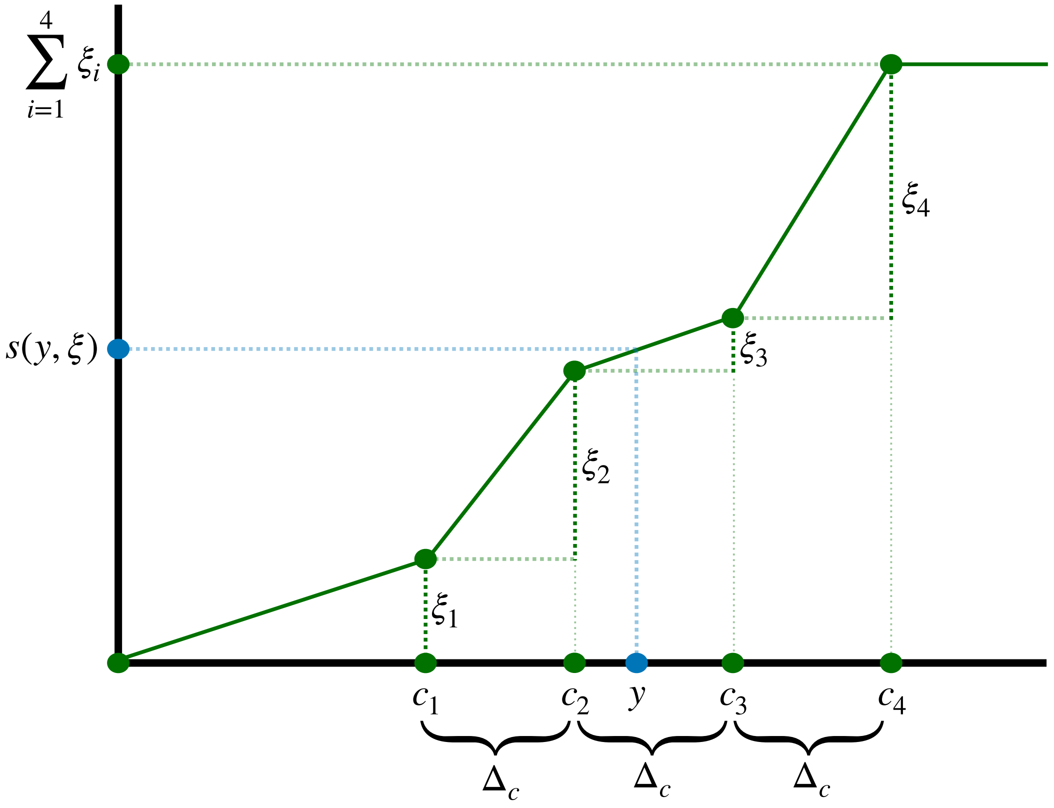

Figure 1: **Illustration of the spline definition notation.**

approximated this non-linear mapping using a piecewise linear spline $s : \mathbb{R} \times \mathbb{R}^{n_\xi} \to \mathbb{R}$, where n_ξ is the number of spline knots. The spline is given by

$$s(y,\xi) := \begin{cases} \frac{y}{c_1} \cdot \xi_1, & y \leq c_1 \\ \frac{y-c_{j-1}}{\Delta_c}\xi_j + \sum_{l=1}^{j-1} \xi_l, & c_{j-1} \leq y \leq c_j \\ \xi_{n_\xi}, & y > c_{n_\xi} \end{cases} \tag{3}$$

in which ξ are the differences between the heights of neighboring spline knots (Figure 1). In other words, the height of the i -th spline knot is equal to $\sum_{j=1}^{i} \xi_j$. With $(c_j)_{j=1}^{n_\xi}$, we denote the uniformly distributed bases of the spline knots, that is, $c_j = c_1 + (j-1) \cdot \Delta_c$. In application, the true range of the biochemical or epidemiological quantities of interest is generally not known a priori. Thus, it is impossible to correctly fix the spline interval $[c_1, c_{n_\xi}]$. However, since we will use iterative optimization methods, at each optimization iteration, we will have access to the simulated quantities of interest $(y_k)_{k=1}^{n_t}(\theta)$. Therefore, to avoid this problem, we always scale the spline interval to the current model output interval $\left[\min_k y_k(\theta), \max_k y_k(\theta)\right]$. This makes the spline parameter bases dependent on the quantities of interest.

$$\begin{aligned} c_1(\theta) &= \min_k y_k(\theta) \\ \Delta_c(\theta) &= \frac{1}{n_\xi - 1}\left(\max_k y_k(\theta) - \min_k y_k(\theta)\right) \\ c_j(\theta) &= c_1(\theta) + (j-1) \cdot \Delta_c(\theta) \end{aligned} \tag{4}$$

However, this does not cause any issues other than slightly increasing the complexity of the analytical gradient computation.

1.2 Objective function and the hierarchical optimization problem

The spline links the measured quantities to the quantities of interest of the non-linear measured observable

$$\tilde{z}_k = s(h(x(t_k,\theta),\theta),\xi) + \varepsilon_k, \quad \text{with } \varepsilon_k \sim \mathcal{N}(0,\sigma_k^2)$$

and thus yields the negative log-likelihood function

$$\begin{aligned} J(\theta,\sigma,\xi) = -\log \mathcal{L}_\mathcal{D}(\theta,\xi) &= \frac{1}{2}\sum_{k=1}^{n_t} \log(2\pi\sigma_k^2) + \frac{1}{\sigma_k^2}\left(\tilde{z}_k - s(y_k(\theta),\xi)\right)^2 \\ &= \frac{1}{2}\sum_{k=1}^{n_t} \log(2\pi\sigma_k^2) + \frac{1}{\sigma_k^2}\left(\tilde{z}_k - \frac{y_k - c_{n(k)-1}}{\Delta_c}\xi_{n(k)} - \sum_{l=1}^{n(k)-1}\xi_l\right)^2 \end{aligned} \tag{5}$$

for a dataset $\mathcal{D} = \{(t_k, \tilde{z}_k)\}_{k=1}^{n_t}$ and simulated observables $y_k = h(x(t_k,\theta),\theta)$, for $k = 1,\dots,n_t$. Here, $(n(k))_{k=1}^{n_t}$ denote subinterval indices for simulated quantities of interest, i.e. $y_k \in [c_{n(k)-1}, c_{n(k)}]$ for $k = 1,\dots,n_t$. We note that currently the objective function depends on the mechanistic parameters θ only through simulations $y_k(\theta)$. The objective function is optimized hierarchically in three nested levels:

$$\min_\theta \; J(\theta,\sigma^*(\theta),\xi^*(\theta)) \tag{6}$$

$$\text{s.t.} \begin{cases} \xi^*(\theta) = \arg\min_\xi J(\theta,\sigma^*(\theta),\xi) \\ \text{s.t. } \xi \geq 0 \text{ and } \begin{cases} \sigma^*(\theta) = \arg\min_\sigma J(\theta,\sigma,\xi) \\ \text{s.t. } \sigma \geq 0. \end{cases} \end{cases} \tag{7}$$

in which the mechanistic parameters are optimized in the outer optimization problem (6), whereas the spline parameters and noise parameters are optimized in two nested inner optimization problems (7). The true measurement mapping is assumed to be monotone, so the spline parameters are constrained to be positive. This is sufficient even in the case of a decreasing true measurement mapping, as inverting the sign of the quantities of interest will turn it into an increasing one. The main benefit of the hierarchical optimization framework is the simplicity of the inner problems. The inner optimization problem for noise parameters σ can be solved analytically, yielding

$$\begin{aligned} 0 = \frac{\partial J}{\partial \sigma_I}(\theta,\sigma^*,\xi) &= \frac{1}{2}\sum_{i\in I}\left(\frac{1}{2\pi(\sigma_I^*)^2}\cdot 2\pi 2\sigma_I^* - \frac{2}{(\sigma_I^*)^3}\left(\tilde{z}_k - s(y_k(\theta),\xi)\right)^2\right) \\ \Rightarrow \sigma_I^* &= \sqrt{\frac{1}{|I|}\sum_{i\in I}\left(\tilde{z}_k - s(y_k(\theta),\xi)\right)^2}. \end{aligned} \tag{8}$$

in which by $I \subset \{1,\dots,n_t\}$ we denote the subset of indices that share the noise parameter σ_I. The σ non-negativity constraint is satisfied as this analytical result already provides a non-negative value. The inner problem for the spline parameters cannot be solved analytically, but we prove that it is

convex in the following theorem. Therefore, its numerical optimization is computationally efficient.

We claim that the defined objective function is convex. We will prove this using the following theorem on the convexity of any least-squares optimization problem with non-negativity constraints.

Theorem 1. *Let an objective function $L \in C^2(\mathbb{R}^{n_\xi}, \mathbb{R})$ be of the form*

$$L(\xi) = \|\tilde{Z} - Y\xi\|_2^2 + b = \|\tilde{Z}\|_2^2 - 2\tilde{Z}^T Y\xi + (Y\xi)^T Y\xi + b \tag{9}$$

where $\tilde{Z} \in \mathbb{R}_t^n$, $Y \in \mathbb{R}^{n_t \times n_\xi}$, $b \in \mathbb{R}$, and $\|\cdot\|_2$ is the L2 norm. Then the optimization problem

$$\begin{cases} \xi^* = \arg\min_{\xi} L(\xi) \\ s.t.\ \ \xi \geq 0 \end{cases} \tag{10}$$

is convex.

Proof. We show that the Hessian of this function is positive semi-definite. The gradient of the objective function (9) is

$$\nabla_\xi J(\xi) = -2\tilde{Z}^T Y + 2\xi^T Y^T Y. \tag{11}$$

Hence, the Hessian matrix of the objective function at the point ξ is given by the Jacobian of the gradient

$$H_J(\xi) = D_\xi(\nabla_\xi J)(\xi) = 2Y^T Y. \tag{12}$$

This is a positive semi-definite matrix since, for any vector $u \in \mathbb{R}^{n_\xi}$, the following holds:

$$u^T H_J(\xi) u = 2u^T Y^T Y u = 2(Yu)^T (Yu) = 2\sum_{i=1}^{n_\xi} (Yu)_i^2 \geq 0 \tag{13}$$

where $(Yu)_i$ is the i-th element of the vector Yu. In combination with affine inequality constraints, this implies that the optimization problem is convex. □

The negative log-likelihood function defined in (5) can be easily reformulated into the form (9), where $\tilde{Z}$, Y, and b can depend on θ and σ but not on ξ. Thus, the inner optimization problem for the spline parameters is convex.

1.3 Analytical gradient

Due to the dependency of the optimal spline parameters ξ^* and optimal noise parameters σ^* on the mechanistic parameters θ, the derivative of the objective function with respect to a mechanistic parameter θ_i contains two additional terms

$$\frac{\partial}{\partial \theta_i} J(\theta, \sigma^*(\theta), \xi^*(\theta)) = \sum_{k=1}^{n_t} \frac{\partial J}{\partial y_k}(\theta, \sigma^*(\theta), \xi^*(\theta)) \cdot \frac{\partial y_k}{\partial \theta_i}(\theta) \tag{14}$$

$$+ \sum_{k=1}^{n_t} \underbrace{\frac{\partial J}{\partial \sigma_k^*}(\theta, \sigma^*(\theta), \xi^*(\theta))}_{=0} \cdot \frac{\partial \sigma_k^*}{\partial \theta_i}(\theta) \tag{15}$$

$$+ \sum_{j=1}^{n_\xi} \underbrace{\frac{\partial J}{\partial \xi_j^*}(\theta, \sigma^*(\theta), \xi^*(\theta)) \cdot \frac{\partial \xi_j^*}{\partial \theta_i}(\theta)}_{=0} \tag{16}$$

where we refer to $\frac{\partial y_k}{\partial \theta_i}$ as the observable sensitivity at time point t_k with respect to the parameter θ_i. The first gradient term can be calculated using forward sensitivity analysis (FSA) or adjoint sensitivity analysis (ASA). Furthermore, the inner problem with respect to the noise parameters is solved exactly. Therefore, the second term (15) is always 0. This leaves the third term to be obtained. In the following theorem, we prove that it is always equal to 0 as well. The theorem is equivalent to the envelope theorem, which is used mainly in economic theory [Carter, 2001].

Theorem 2. *For any $\theta \in \mathbb{R}^{n_\theta}$, let the pair $(\eta^*(\theta), \phi^*(\theta)) \in \mathbb{R}^{n_\eta \times n_\phi}$ be a solution of the following optimization problem*

$$\begin{cases} (\eta^*(\theta), \phi^*(\theta)) = \arg\min_{\eta,\phi} J(\theta, \eta, \phi) \\ s.t.\ \ \phi \geq 0 \end{cases} \tag{17}$$

where $J \in C^2(\mathbb{R}^{n_\theta \times n_\eta \times n_\phi}, \mathbb{R})$ is a convex objective function and ϕ is constrained to be positive. Then for all $\theta \in \mathbb{R}^{n_\theta}$, $i = 1, \dots, n_\theta$, and $j = 1, \dots, n_\phi$, $k = 1, \dots, n_\eta$

$$\frac{\partial J}{\partial \eta_k}(\theta, \eta^*(\theta), \phi^*(\theta)) = 0 \tag{18}$$

$$\frac{\partial J}{\partial \phi_j}(\theta, \eta^*(\theta), \phi^*(\theta)) \cdot \frac{\partial \phi_j^*}{\partial \theta_i}(\theta) = 0 \tag{19}$$

Proof. The pair $(\eta^*(\theta), \phi^*(\theta))$ is a solution to a convex optimization problem with affine inequality constraints. Therefore, Slater's condition holds [Slater, 1959], and there exist optimal Lagrange multipliers $\mu^*(\theta) = (\mu_i^*(\theta))_{i=1}^{n_\phi}$ such that the triplet $(\eta^*(\theta), \phi^*(\theta), \mu^*(\theta))$ satisfies the Karush-Kuhn-Tucker (KKT) conditions

$$\nabla_\eta J(\theta, \eta^*(\theta), \phi^*(\theta)) = 0 \tag{20}$$

$$\nabla_\phi J(\theta, \eta^*(\theta), \phi^*(\theta)) - \mu^*(\theta) = 0 \tag{21}$$

$$\mu_j^* \phi_j^* = 0, \quad \text{for } j = 1, \dots, n_\phi \tag{22}$$

$$\phi_j^* \geq 0, \quad \text{for } j = 1, \dots, n_\phi$$

$$\mu_j^* \geq 0, \quad \text{for } j = 1, \dots, n_\phi.$$

The first KKT condition (20) directly proves the first statement of the Theorem (18). Furthermore, due to the simple positivity constraints, the second KKT condition (21) states that the Lagrange multipliers are equal to the objective function gradient with respect to ϕ

$$\mu_j^*(\theta) = \frac{\partial J}{\partial \phi_j^*}(\theta, \eta^*(\theta), \phi^*(\theta)) \quad \text{for } j = 1, \dots, n_\phi. \tag{23}$$

To prove the first statement of the theorem (18) for an index $j \in \{1, \dots, n_\phi\}$, we consider two cases: $\phi_j^* > 0$ and $\phi_j^* = 0$.
Case 1: $\phi_j^* > 0$. From the third KKT condition (22), we then know that $\mu_j^*(\theta) = 0$. Therefore, the statement follows from (23).
Case 2: $\phi_j^* = 0$. For an index $i \in \{1, \dots, n_\theta\}$, we differentiate the third KKT condition (22) with respect to θ_i to obtain

$$0 = \frac{\partial \mu_j^*}{\partial \theta_i} \underbrace{\phi_j^*}_{=0} + \frac{\partial \phi_j^*}{\partial \theta_i} \mu_j^* \overset{(23)}{=} \frac{\partial \phi_j^*}{\partial \theta_i} \cdot \frac{\partial J}{\partial \phi_j^*}(\theta, \eta^*(\theta), \phi^*(\theta))$$

□

Since the negative log-likelihood function defined in (5) is convex it satisfies the theorem's conditions. In addition, in this case, $\eta = \sigma$ and $\phi = \xi$. Therefore, the derivative of the objective function with respect to a mechanistic parameter θ_i can be analytically computed and is equal to

$$\frac{\partial}{\partial \theta_i} J\left(\theta, \sigma^*(\theta), \xi^*(\theta)\right) = \sum_{k=1}^{n_t} \frac{\partial J}{\partial y_k}\left(\theta, \sigma^*(\theta), \xi^*(\theta)\right) \cdot \frac{\partial y_k}{\partial \theta_i}(\theta) \tag{24}$$

1.4 Spline regularization

In our implementation, the spline (3) is additionally regularized to reduce overfitting. The regularization penalizes the non-linearity of the spline with the aim of minimizing false-positive non-linear measurement mappings. To achieve this, we add the following regularization term to the objective function:

$$R(\xi) = \frac{1}{2n_\xi} \|\xi - \alpha^*(\xi) \cdot c - \beta^*(\xi)\|_2^2 \tag{25}$$

where

$$\begin{aligned} \alpha^*(\xi), \beta^*(\xi) = \arg\min_{\alpha,\beta} \|\xi - \alpha \cdot c - \beta\|_2^2 \\ \text{s.t. } \beta^*(\xi) \geq 0. \end{aligned} \tag{26}$$

In other words, $\alpha^*(\xi)$ and $\beta^*(\xi)$ are the optimal scaling and offset of a linear regression of the spline knots $\{(c_j, \xi_j)\}_{j=1}^{n_\xi}$ and the regularization is a penalization of the distance between the spline knots and the optimal linear line. Having a negative offset is not plausible in most applications. Thus, we constrain the offset to be non-negative.

We will show this optimization problem can be solved analytically. To do so, we state and prove the following simple proposition.

Proposition. *Let $(\bar{\alpha}, \bar{\beta})$ be the unique solution of an unconstrained optimization problem*

$$\bar{\alpha}, \bar{\beta} = \arg\min_{\alpha,\beta} L(\alpha, \beta) \tag{27}$$

where $L : \mathbb{R}^2 \to \mathbb{R}$ is a strictly convex objective function. Then the unique solution (α^*, β^*) of the optimization problem with a positivity constraint

$$\begin{aligned} \alpha^*, \beta^* = \arg\min_{\alpha,\beta} L(\alpha, \beta) \\ s.t. \ \beta \geq 0 \end{aligned} \tag{28}$$

satisfies the following:

$$\beta^* = \begin{cases} \bar{\beta} & \text{if } \bar{\beta} \geq 0 \\ 0 & \text{otherwise} \end{cases} \tag{29}$$

In other words, we can solve for the positivity-constrained variable by simply solving the unconstrained problem and inspecting whether it is positive. If it is, then the solution is given by it. Otherwise, the optimal solution is on the constraint, i.e. 0.

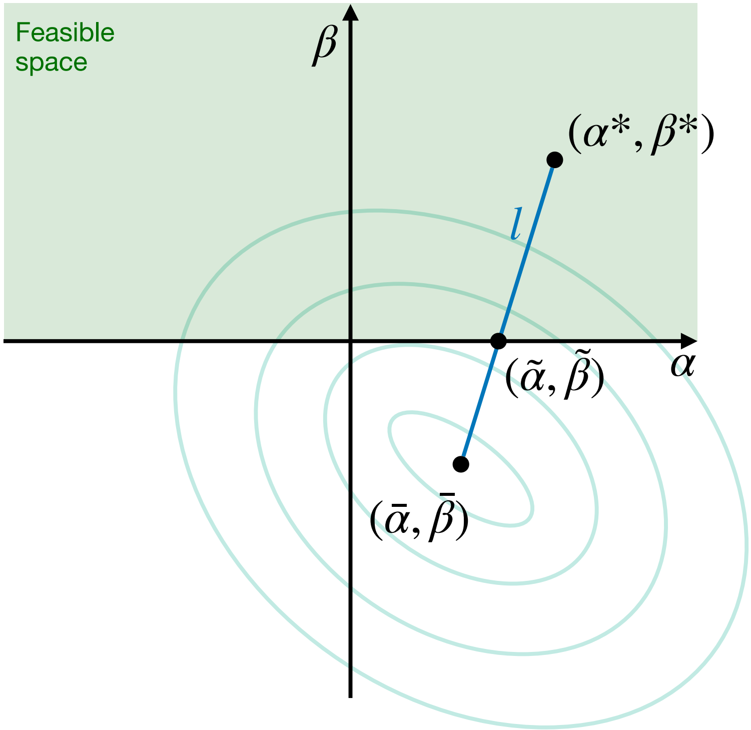

Figure 2: **Proposition figure.**

Proof. The first statement of the proposition is trivial. If the solution of the unconstrained problem lies in the feasible region of the constrained problem, then the solutions $(\bar{\alpha}, \bar{\beta})$ and (α^*, β^*) are equal due to the same strictly convex objective function of the optimization. To prove the second statement, let us assume the opposite: $\bar{\beta} < 0$, but $\beta^* > 0$. Then we connect the two solutions by a line in the two-dimensional parameter space $l : [0, 1] \to \mathbb{R}^2$ from (α^*, β^*) to $(\bar{\alpha}, \bar{\beta})$: $l(x) = (1 - x) \cdot (\alpha^*, \beta^*) + x \cdot (\bar{\alpha}, \bar{\beta})$. Since these points are on opposite sides of the constraint, the line will cross the constraint at some point $(\tilde{\alpha}, \tilde{\beta})$. This point also satisfies $J(\alpha^*, \beta^*) < J(\tilde{\alpha}, \tilde{\beta})$ because it lies in the feasible parameter space and is not equal to the optimal point (α^*, β^*) due to being on the constraint $\beta^* > 0 = \tilde{\beta}$. However, from the solution of the unconstrained problem, we know that $J(\tilde{\alpha}, \tilde{\beta}) > J(\bar{\alpha}, \bar{\beta})$. In conclusion, we have found a line l with three points on it that satisfy $J(\alpha^*, \beta^*) < J(\tilde{\alpha}, \tilde{\beta}) > J(\bar{\alpha}, \bar{\beta})$. This is in contradiction to the strict convexity of the function J and therefore the second statement holds as well. □

Now we will apply the proposition to our problem. The optimization problem (26) satisfies the conditions of Theorem (1), so it is convex. Furthermore, it is strictly convex due to the full rank of the corresponding Hessian matrix $Y^T Y$, which makes the inequality (13) strict. Thus, it satisfies the conditions of the proposition. To obtain the solution of the unconstrained version of the problem, we equate the gradient of the objective function to 0. This gives a linear system whose solution is

$$\bar{\beta}(\xi) = \frac{\sum_{j=1}^{n_\xi} \xi_j \cdot \sum_{j=1}^{n_\xi} c_j^2 - c \cdot \xi \cdot \sum_{j=1}^{n_\xi} c_j}{n_\xi \cdot \sum_{j=1}^{n_\xi} c_j^2 - \left(\sum_{j=1}^{n_\xi} c_j\right)^2}$$

$$\bar{\alpha}(\xi) = \frac{c \cdot \xi - \bar{\beta}(\xi) \cdot \sum_{j=1}^{n_\xi} c_j}{\sum_{j=1}^{n_\xi} c_j^2}.$$

According to the proposition, to obtain the solution to the unconstrained problem we inspect the sign of $\bar{\beta}(\xi)$. If it is positive, the solution is equal to $(\bar{\alpha}(\xi), \bar{\beta}(\xi))$. Otherwise, the optimal $\beta^*(\xi)$ is equal to 0. To obtain the optimal scaling $\alpha^*(\xi)$ for this case we turn to the KKT conditions of the constrained optimization problem:

$$\frac{\partial}{\partial \alpha} \|\xi - \alpha^* \cdot c - \beta^*\|_2^2 = 0 \tag{30}$$

$$\frac{\partial}{\partial \beta} \|\xi - \alpha^* \cdot c - \beta^*\|_2^2 - \mu^* = 0 \tag{31}$$

$$\mu^* \beta^* = 0 \tag{32}$$

$$\mu^* \geq 0 \tag{33}$$

$$\beta^* \geq 0 \tag{34}$$

where $\mu^* \in \mathbb{R}$ is the optimal Lagrange multiplier. The first KKT condition (30) is a linear equation in $\alpha^*(\xi)$ whose solution for $\beta^*(\xi) = 0$ is

$$\alpha^*(\xi) = \frac{c \cdot \xi}{\sum_{j=1}^{n_\xi} c_j^2}.$$

Pulling the two cases together, we can write the final analytical solution to the optimization problem as

$$\bar{\beta}(\xi) = \frac{\sum_{j=1}^{n_\xi} \xi_j \cdot \sum_{j=1}^{n_\xi} c_j^2 - c \cdot \xi \cdot \sum_{j=1}^{n_\xi} c_j}{n_\xi \cdot \sum_{j=1}^{n_\xi} c_j^2 - \left(\sum_{j=1}^{n_\xi} c_j\right)^2}$$

$$\beta^*(\xi) = \begin{cases} \bar{\beta}(\xi) & \text{if } \bar{\beta}(\xi) \geq 0 \\ 0 & \text{otherwise} \end{cases} \tag{35}$$

$$\alpha^*(\xi) = \frac{c \cdot \xi - \beta^*(\xi) \cdot \sum_{j=1}^{n_\xi} c_j}{\sum_{j=1}^{n_\xi} c_j^2} \tag{36}$$

This regularization is appended to the objective function of the model optimization (5). Thus, the complete objective function is given as:

$$\begin{aligned} J(\theta, \sigma, \xi) &= -\log \mathcal{L}_\mathcal{D}(\theta, \sigma, \xi) + \lambda \cdot R(\xi) \\ &= \frac{1}{2} \sum_{k=1}^{n_t} \log(2\pi\sigma_k^2) + \frac{1}{\sigma_k^2} \left(\tilde{z}_k - \frac{y_k - c_{n(k)-1}}{\Delta_c} \xi_{n(k)} - \sum_{l=1}^{n(k)-1} \xi_l \right)^2 \\ &\quad + \frac{\lambda}{2 n_\xi} \|\xi - \alpha^*(\xi) \cdot c - \beta^*(\xi)\|_2^2. \end{aligned} \tag{37}$$

where $\lambda \in \mathbb{R}^+$ is the regularization strength parameter. We note that this addition does not change the result of the analytical gradient (Theorem 2), as the objective function remains convex.

1.5 Models with multiple observables

Here, we show that the results obtained in previous subsections hold for a model with an arbitrary number of semi-quantitative observables n_y. The semi-quantitative data is linked to the observables

via

$$\tilde{z}_{i,k} = s_i(h_i(x(t_k,\theta),\theta),\xi_i) + \varepsilon_{i,k}, \qquad (38)$$
$$\text{with } \varepsilon_{i,k} \sim \mathcal{N}(0,\sigma_{i,k}^2), \text{ for } \quad k = 1,\ldots,n_t, \quad i = 1,\ldots,n_y$$

where each semi-quantitative observable has its own spline function $s_i : \mathbb{R} \times \mathbb{R}^{n_{\xi,i}} \to \mathbb{R}$. We assume that each one of the splines depends on its own vector of spline parameters ξ_i and that these parameters are not shared among observables. Similarly, we assume that the observable noise parameters σ_i are not shared between observables. Thus, the overall objective function J consists of n_y observable-specific objective functions $\{J_i : \mathbb{R}^{n_\theta \times n_t \times n_{\xi,i}} \to \mathbb{R}\}_{i=1}^{n_y}$

$$J(\theta,\sigma,\xi) = \sum_{i=1}^{n_y} J_i(\theta,\sigma_i,\xi_i) = \sum_{i=1}^{n_y} -\log \mathcal{L}_{\mathcal{D}_i}(\theta,\sigma_i,\xi_i) + R_i(\xi_i) \qquad (39)$$

where $\mathcal{D}_i = \{(t_k,\tilde{z}_{i,k})\}_{k=1}^{n_t}$ is the data set of the i-th observable. Therefore, the hierarchical optimization problem contains n_y separate inner sub-problems that can be solved as before.

$$\min_\theta J(\theta,\sigma^*(\theta),\xi^*(\theta)) = \sum_{i=1}^{n_y} J_i(\theta,\sigma_i^*(\theta),\xi_i^*(\theta)) \qquad (40)$$

$$\text{s.t.} \left\{ \begin{array}{l} \xi_i^*(\theta) = \arg\min_\xi J(\theta,\sigma_i^*(\theta),\xi_i) \\ \xi_i^*(\theta) \geq 0. \\ \text{s.t.} \left\{ \sigma_i^*(\theta) = \arg\min_\sigma J(\theta,\sigma_i,\xi_i) \right\} \end{array} \right\} \quad \text{for} \quad i = 1,\ldots,n_y. \qquad (41)$$

To efficiently solve the outer optimization problem, we need to calculate the gradient of the objective function J with respect to the mechanistic parameters θ. Since the objective function is additive in observables, this is given by:

$$\nabla_\theta J(\theta,\sigma^*,\xi^*) = \sum_{i=1}^{n_y} \nabla_\theta J_i(\theta,\sigma_i^*,\xi_i^*) \qquad (42)$$

where $\nabla_\theta J_i(\theta,\sigma_i^*,\xi_i^*)$ can be obtained as before.

2 Model details

For the evaluation of the proposed method, we employed five models in total: one toy model T1 and four published models M1-M4. The models contain a varying number of states, mechanistic parameters, observables, and data points (Table 1). The published models are taken from a collection of parameter estimation problems in PEtab format, which is based on the benchmark collection by Hass et al. [2019].

These models were originally calibrated on quantitative measurements. Thus, to benchmark the proposed method, we modified the observation model to include non-linear measurement mappings. However, as the original model measurements were already noise-corrupt, it is not possible to retain the same noise model by simply applying the non-linear mappings to those measurements. Therefore, we generated synthetic noise-free data and transformed it with the non-linear mappings.

Model	n_x	n_θ	n_y	$\|\mathcal{D}\|$	Description	Reference
T1	2	4	1	12	FRET probe activation	Birtwistle et al. [2011]
M1	8	6	3	48	STAT5 dimerization	Boehm et al. [2014]
M2	7	9	1	23	Infectious disease dynamics	Rahman et al. [2016]
M3	8	18	1	58	Transcriptional regulation	Elowitz and Leibler [2000]
M4	14	18	8	205	IL13-induced signaling	Raia et al. [2011]

Table 1: **Application models.** Consistent with notation from Section 1, n_x, n_θ, and n_y denote the numbers of state variables, mechanistic parameters, and observables, respectively. With $|\mathcal{D}|$ we denote the cardinality of the data set.

Only then could we add noise of the same original magnitude. This resulted in the same noise model as in the original model versions. The mechanistic parameters used to generate the synthetic data were estimated using the original quantitative measurements. These nominal mechanistic parameters are available in the benchmark collection mentioned above.

2.1 Model T1: FRET probe activation

The toy model is a simple model of FRET probe activation introduced by Birtwistle et al. [2011]. The model assumes Michaelis-Menten (MM) mechanisms for both probe activation and deactivation. All mechanistic, noise, and observable parameters are presented in Table 2. The model consists of one differential equation and one conservation law:

$$\frac{\partial P^*}{\partial t} = F_a \cdot \frac{P}{K_{mf} + P} - F_r \frac{P^*}{K_{mr} + P^*} \tag{43}$$

$$P = P_{TOT} - P^*. \tag{44}$$

Furthermore, the model's only observable is the ratiometric imaging intensity ratio

$$R(P^*) = \frac{I_A}{I_D} = \frac{P^* \cdot f_{AA}}{(P_{TOT} - P^*) f_{DD}} + \frac{f_{AD}}{f_{DD}}. \tag{45}$$

Consistent with the notation established in Section 1, the observation function of this observable is the simple identity function $h(P^*) = P^*$, whereas the non-linear measurement mapping g is given by R.

To obtain synthetic data, we simulated the model with the true values of mechanistic parameters from Table 2, applied the measurement mapping function (45), and added additive normal noise with standard deviation given in the same Table.

Three model variants with varying observable models were estimated. The first is the parameterized model. Its observation model was a parameterization of the true measurement mapping (45). To avoid non-identifiability of observable parameters, instead of directly estimating f_{AA}, f_{AD} and f_{DD}, we rather estimated a parameterization with only two observable parameters α and β

$$g_{\text{par}}(P^*) = \alpha \cdot \frac{P^*}{(P_{TOT} - P^*)} + \beta. \tag{46}$$

The second model estimated the measurement mapping using a linear function. Lastly, the third model used the proposed spline estimation method.

Parameter name	Notation	True value	Estimated
Activation enzyme activity	F_a	1	Yes
Deactivation enzyme activity	F_r	1.1	Yes
Activation enzyme MM constant	K_{mf}	0.01	Yes
Deactivation enzyme MM constant	K_{mr}	0.1	Yes
Total probe pool	P_{TOT}	1	No
Standard deviation	σ	0.01	No
Fraction of donor emission captured by donor channel	f_{DD}	0.8	Yes
Fraction of donor emission captured by acceptor channel	f_{AD}	0.3	Yes
Fraction of acceptor emission captured by acceptor channel	f_{AA}	0.8	Yes

Table 2: **Toy model T1 parameters.** The horizontal lines separate mechanistic, noise, and observable parameters, respectively.

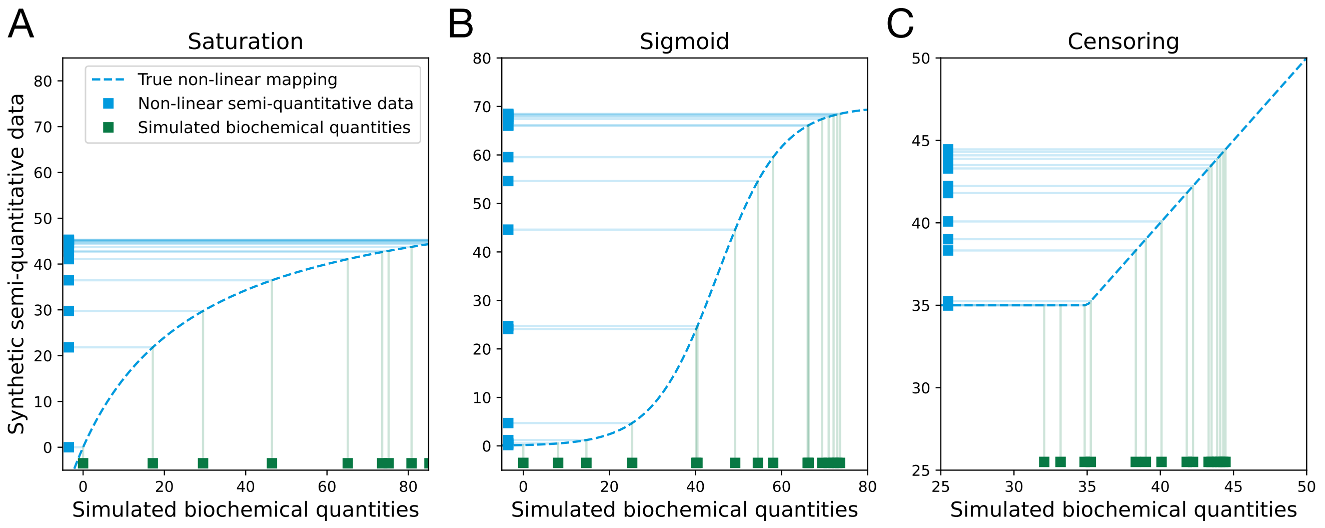

Figure 3: **Synthetic non-linear semi-quantitative data of model M1.** The simulated quantities of interest are denoted on the x-axis in green, whereas the semi-quantitative measurements are denoted on the y-axis in blue. The chosen non-linear measurement mappings are denoted in dashed blue for each observable (A-C).

2.2 Model M1: STAT5 dimerization

The STAT5 dimerization model was introduced by Boehm et al. [2014]. It possesses 3 observables – pSTAT5A_rel, pSTAT5B_rel, and rSTAT5A_rel. We will refer to the observables as first, second, and third in that order. The chosen synthetic non-linear measurement mappings are shown in Figure 3: saturation, sigmoid, and censoring. In the same order, their expressions are

$$g_1(y) = 2 \cdot \frac{y}{1 + \frac{y}{30}} \tag{47}$$

$$g_2(y) = 35 \cdot \tanh\left(\frac{y}{15} - 3\right) + 35 \tag{48}$$

$$g_3(y) = \begin{cases} y, & \text{if } y > 35 \\ 35, & \text{otherwise} \end{cases} \tag{49}$$

where the specific numerical values were chosen such that the mappings are significantly non-linear in the range of the respective simulated biochemical quantities.

2.3 Model M2: Infections disease dynamics

The model of infectious disease dynamics was introduced by Rahman et al. [2016]. It describes the impacts of early treatment programs on HIV epidemics and overall community-level immunity. It possesses only one observable, HIV prevalence. The chosen non-linear measurement mapping is a hyperbolic growth function

$$g(y) = \frac{1}{1 - \frac{y}{19}} \tag{50}$$

where the specific parameterization was chosen such that the mapping is significantly non-linear in the range of the simulated quantity of interest.

2.4 Model M3: Transcriptional regulation

The model of transcriptional regulation was introduced by Elowitz and Leibler [2000]. It models an oscillating network consisting of three transcriptional repressor systems, termed the repressilator, in Escherichia coli. It possesses only one observable – protein fluorescence readout. The chosen non-linear measurement mapping is a saturation function

$$g(y) = 10 \tanh(y) \tag{51}$$

where the specific parameterization was chosen such that the mapping is significantly non-linear in the range of the simulated quantity of interest.

2.5 Model M4: IL13-induced signaling

The model of IL13-induced JAK2/STAT5 pathway signaling in lymphoma cell lines was introduced by Raia et al. [2011]. It possesses 8 observables. We chose non-linear measurement mappings of the same shape across observables, scaled to the range of the simulated quantities of interest. The functions are

$$g_i(y) = a_i \cdot \tanh\left(\frac{y}{a_i}\right), \quad \text{for } i = 1, \dots, 8 \tag{52}$$

where $(a_i)_{i=1}^{8} = (66, 0.66, 0.45, 81, 450, 0.24, 0.78, 31.5)$ are the scaling factors chosen such that the mappings are significantly non-linear in the range of the respective simulated quantities of interest.

3 Implementation and optimization details

The proposed method is implemented in the open-source Python Parameter Estimation TOolbox (pyPESTO, Schälte et al. [2023]). The implementation allows for the specification of an arbitrary number of spline knots and spline regularization strength. Within the GitHub repository of pyPESTO, there is a Jupyter notebook available that showcases the usage of the method.

Parameter estimation of all published models was performed using multi-start local optimization with 1000 starts per model. Each estimation was run on 10 cores of the AMD EPYC 7F72 3.20 GHz processor with 1TB of RAM. Gradient-based optimization was performed using the fides optimizer [Fröhlich and Sorger, 2022] and gradient-free optimization was performed with the SciPy Powell

algorithm [Jones et al., 2001]. Both optimizers were accessed through the pyPESTO interface with the default optimizer settings. For optimization of inner problems, we used SciPy's L-BFGS-B algorithm with default settings. For ODE integration, we used the AMICI Python toolbox [Fröhlich et al., 2021].

4 Parameter inference study

In the last subsection of the Results section in the manuscript, we presented a study focused on parameter inference. We compared the linear estimation and spline estimation approaches in the four application examples M1 - M4 with their respective synthetic non-linear measurement mappings. Furthermore, we evaluated the impact of an increasing number of unknown measurement mappings.

For illustration, we consider the model M1. It contains three observables, each with its own non-linear measurement mapping (Fig. 3). However, since these mappings are synthetic and thus known, we were able to include the mappings $\{g_i\}_{i=1}^3$ in the observable function $\{h_i\}_{i=1}^3$, transforming the semi-quantitative observables into standard quantitative ones. This is how we obtained model variants of the same initial model M1 with a variable number of unknown measurement mappings. Furthermore, to remove any observable bias, we considered all combinations of unknown and known observables (Table 3).

Model variant # / Observable #	1	2	3	# unknown observables
1	●	●	●	0
2	○	●	●	1
3	●	○	●	1
4	●	●	○	1
5	○	○	●	2
6	○	●	○	2
7	●	○	○	2
8	○	○	○	3

Table 3: **Observable combinations of model M1.** ● and ○ denote whether the measurement mapping of the observable is known or unknown, respectively.

For models M2 and M3 this is simple, as they contain only one observable so we considered two variants: known observable mapping and unknown observable mapping. Model M4 contains 8 observables, so it would have been computationally too expensive to include all $2^8 = 256$ model variants with 3 estimation approaches. Therefore, to reduce the observable bias while keeping the number of model variants reasonably low, we considered the model M4 variants shown in Table 4.

Model variant # / Observable #	1	2	3	4	5	6	7	8	# unknown observables
1	●	●	●	●	●	●	●	●	0
2	○	●	●	●	●	●	●	●	1
3	○	○	●	●	●	●	●	●	2
4	○	○	○	●	●	●	●	●	3
5	○	○	○	○	●	●	●	●	4
6	○	○	○	○	○	●	●	●	5
7	○	○	○	○	○	○	●	●	6
8	○	○	○	○	○	○	○	●	7
9	●	●	●	●	●	●	●	○	1
10	●	●	●	●	●	●	○	○	2
11	●	●	●	●	●	○	○	○	3
12	●	●	●	●	○	○	○	○	4
13	●	●	●	○	○	○	○	○	5
14	●	●	○	○	○	○	○	○	6
15	●	○	○	○	○	○	○	○	7
16	○	●	●	●	●	●	●	●	1
17	○	●	●	●	●	●	●	○	2
18	○	○	●	●	●	●	●	○	3
19	○	○	●	●	●	●	○	○	4
20	○	○	○	●	●	●	○	○	5
21	○	○	○	●	●	○	○	○	6
22	○	○	○	○	●	○	○	○	7
23	○	○	○	○	○	○	○	○	8

Table 4: **Observable combinations of model M4.** ● and ○ denote whether the measurement mapping of the observable is known or unknown, respectively.

References

M. R. Birtwistle, A. von Kriegsheim, K. Kida, J. P. Schwarz, K. I. Anderson, et al. Linear approaches to intramolecular förster resonance energy transfer probe measurements for quantitative modeling. *PLOS ONE*, 6(11):e27823, 2011.

M. E. Boehm, L. Adlung, M. Schilling, S. Roth, U. Klingmueller, et al. Identification of isoform-specific dynamics in phosphorylation-dependent stat5 dimerization by quantitative mass spectrometry and mathematical modeling. *Journal of Proteome Research*, 13(12):5685–5694, 2014.

M. Carter. *Foundations of Mathematical Economics*, volume 1 of *MIT Press Books*. The MIT Press, December 2001. ISBN ARRAY(0x342691a0). URL `https://ideas.repec.org/b/mtp/titles/0262032899.html`.

M. B. Elowitz and S. Leibler. A synthetic oscillatory network of transcriptional regulators. *Nature*, 403(6767):335–338, 2000.

F. Fröhlich and P. K. Sorger. Fides: Reliable trust-region optimization for parameter estimation of ordinary differential equation models. *PLoS Comput Biol*, 18(7):1–28, 07 2022. doi: 10.1371/journal.pcbi.1010322. URL `https://doi.org/10.1371/journal.pcbi.1010322`.

F. Fröhlich, D. Weindl, Y. Schälte, D. Pathirana, Paszkowski, et al. AMICI: high-performance sensitivity analysis for large ordinary differential equation models. *Bioinformatics*, 37(20):3676–3677, 04 2021. ISSN 1367-4803. doi: 10.1093/bioinformatics/btab227.

H. Hass, C. Loos, E. Raimúndez-Álvarez, J. Timmer, J. Hasenauer, et al. Benchmark problems for dynamic modeling of intracellular processes. *Bioinformatics*, 35(17):3073–3082, 01 2019. ISSN 1367-4803. doi: 10.1093/bioinformatics/btz020.

E. Jones, T. Oliphant, P. Peterson, et al. SciPy: Open source scientific tools for Python, 2001. URL `http://www.scipy.org/`.

S. M. A. Rahman, N. K. Vaidya, and X. Zou. Impact of early treatment programs on hiv epidemics: An immunity-based mathematical model. *Mathematical biosciences*, 280:38—49, October 2016. ISSN 0025-5564. doi: 10.1016/j.mbs.2016.07.009. URL `https://doi.org/10.1016/j.mbs.2016.07.009`.

V. Raia, M. Schilling, M. Böhm, B. Hahn, A. Kowarsch, et al. Dynamic mathematical modeling of il13-induced signaling in hodgkin and primary mediastinal b-cell lymphoma allows prediction of therapeutic targets. *Cancer research*, 71(3):693–704, 2011.

Y. Schälte, F. Fröhlich, P. J. Jost, J. Vanhoefer, D. Pathirana, et al. pyPESTO: A modular and scalable tool for parameter estimation for dynamic models. *Bioinformatics*, page btad711, 11 2023. ISSN 1367-4811. doi: 10.1093/bioinformatics/btad711. URL `https://doi.org/10.1093/bioinformatics/btad711`.

M. Slater. Lagrange multipliers revisited. (80), 1959. URL `https://EconPapers.repec.org/RePEc:cwl:cwldpp:80`.
